# Pathways of rDNA copy number homeostasis in *Schizosaccharomyces pombe*

**DOI:** 10.1093/g3journal/jkag093

**Published:** 2026-04-28

**Authors:** Chance E Jones, Ji-ping Yuan, Susan L Forsburg

**Affiliations:** Molecular & Cellular Biosciences, University of Southern California, 1050 Childs Way, RRI 108, Los Angeles, CA 90089, United States; Molecular & Cellular Biosciences, University of Southern California, 1050 Childs Way, RRI 108, Los Angeles, CA 90089, United States; Molecular & Cellular Biosciences, University of Southern California, 1050 Childs Way, RRI 108, Los Angeles, CA 90089, United States

**Keywords:** rDNA copy number, genome stability, *Schizosaccharomyces pombe*, DNA replication stress, DDK kinase, fork protection complex, alkylation damage, repetitive DNA

## Abstract

Fragile sites across the genome pose an increased risk of genetic instability. The rDNA repeats are particularly at risk due to highly repetitive sequences, replication transcription collisions, polar replication fork barriers, and late DNA replication. In this study, we examine mechanisms of rDNA homeostasis in fission yeast. We monitor the effects of an artificially contracted rDNA array, assess the responses to genome-wide alkylation damage, and identify genetic pathways that affect rDNA copy number. We find that a reduced rDNA array leads to a decreased growth rate, smaller cell size, and increased genotoxic sensitivity to alkylation damage from MMS. We also observe that in response to chronic MMS exposure, normal rDNA arrays contract in a time/concentration-dependent manner. We show that rDNA copy number is affected by the fork protection complex (FPC), fork licensing proteins, chromatin modifiers, and DDK kinase. These results confirm that the rDNA repeats are a genome fragile site that is particularly sensitive to perturbations in DNA replication.

## Teaser

This study explores how cells maintain the correct number of ribosomal DNA (rDNA) repeats, which are crucial for making ribosomes and supporting cell growth. Using fission yeast as a model, Jones *et al*. identified genetic pathways that either protect or destabilize rDNA. The authors showed that having too few rDNA copies causes slower growth, smaller cells, and sensitivity to DNA damage. They also found that certain stress conditions and mutations cause rDNA loss over time. These results highlight the importance of rDNA copy number in genome stability and help explain how cells adapt to replication stress.

## Introduction

DNA replication stress can be caused by intrinsic genomic features that impede the smooth progression of the replication fork (Magdalou et al. 2014; Zeman and Cimprich 2014). In particular, repetitive DNA sequences may create chromosome fragile sites that are associated with breaks and genome instability (Debatisse et al. 2012; Glover et al. 2017). Previously, we showed that the repetitive heterochromatic pericentromere is associated with replication stress-induced breaks and rearrangements when heterochromatin is impaired (Li et al. 2013), establishing this as a model fragile site that is protected by heterochromatin. In this study, we examine another repetitive sequence domain, the ribosomal DNA.

The ribosomal DNA (rDNA) encodes the ribosomal RNA genes and resides in the nucleolus, a sub-nuclear domain in eukaryotic cells. Due to the high demand for ribosome biogenesis, the rDNA is organized into repeats that form high-copy-number arrays. In the fission yeast *Schizosaccharomyces pombe,* there are roughly ∼150 repeats distributed between the 2 telomere proximal arrays at the ends of Chromosome III (Toda et al. 1984). Approximately half of these repeats are actively transcribed while the other are heterochromatinized in actively dividing WT cells (French et al. 2003; Ide et al. 2010). The rDNA repeats code for the highly transcribed pre-rRNA sequence and are then subsequently processed in the nucleolus to the 18s, 5.8s, and 28s rRNA before nucleolar export (Good et al. 1997). Transcription of these rRNAs makes up roughly 60% of the eukaryotic transcriptome (Ide et al. 2010). Along with the coding regions, each array also contains an intergenic sequence (IGS) consisting of a replication fork barrier (RFB) and a single origin of replication or autonomously replicating sequence (ARS) (Shirahige et al 1998; Kim and Huberman 2001; Kobayashi and Ganley 2005). The RFB is targeted by specific proteins to ensure unidirectional DNA replication (Jaiswal et al. 2016), which helps avoid replication transcription collisions in this highly transcribed domain.

The RFB mechanism varies among eukaryotes. In *Saccharomyces cerevisiae,* the Fob1 protein binds to the RFB, causing an abrupt replication fork block, and is also essential for rDNA recombination (Kobayashi 2003). In *S. pombe,* there is a quaternary RFB system with several *ter*, or termination sites. The first Ter site is bound by the Sap1 protein, and Ter2 and 3 are bound by the Reb1 protein (Singh et al. 2010). RFB4 is not protein-associated, but is located in the terminal transcription region of the preceding 35s rDNA (Sánchez-Gorostiaga et al. 2004; Krings and Bastia 2005). This system is similar to higher eukaryotes, which also have multiple pausing sites at each RFB (Akamatsu and Kobayashi 2015). Components of the fork protection complex (FPC), including Swi1/Swi3 and Mrc1 (Tof1/Csm3 and Mrc1 in *S. cerevisiae*), are essential for proper fork pausing (Krings and Bastia 2005). However, while Swi1/3 are essential for RFB pausing, loss of Mrc1 only causes a 26% decrease in RFB pausing (Krings and Bastia 2005; Zech et al. 2015). With these proteins, the cell mitigates the toxicity of replication transcription collisions on the one hand and replication fork collapse from stalled forks on the other (Brewer and Fangman 1988; Tsang and Carr 2008).

Cells have developed multiple mechanisms to maintain a proper rDNA copy number homeostasis (Nelson et al. 2019). The most well-known and studied is via cohesin restraining neighboring sister chromatid rDNA repeats, which restricts unequal sister chromatid exchange (USCE) in *S. cerevisiae* (Kobayashi and Ganley 2005). Another mechanism maintaining rDNA copy number homeostasis is large intrachromatid recombination, most often leading to extrachromosomal rDNA circles (ERCs) (Sinclair and Guarente 1997). These can undergo excision and reinsertion into the genome, creating large changes in rDNA chromosomal copy number without much effect on rRNA output. This process of ERC formation and rDNA excision has also been correlated with limited replicative aging in yeast (Sinclair and Guarente 1997; Kobayashi 2011; Hotz et al. 2022). A third mechanism maintaining proper rDNA copy number homeostasis is that of heterochromatin formation, silencing unused rDNA repeats (Srivastava et al. 2016). It has been proposed that heterochromatin formation restricts transcription replication collisions, thus stabilizing unused repeats. In *Drosophila* mutants lacking the main heterochromatin binding protein HP1, there is a gradual decrease in rDNA repeat copy number (Lawrence et al. 2004; Pontvianne et al. 2012). Overall, cells rely on various mechanisms to respond to high or low rDNA copy numbers to maintain a particular homeostatic copy number. However, these mechanisms may not be able to cope with genome instability, thus leading to an increase or decrease in rDNA copy number away from homeostasis. In budding yeast, studies suggest reduced or excessive rDNA copy number leads to increased genome instability (Ide et al. 2010; Kwan et al. 2016; Kwan et al. 2023), and these can help or hinder various environmental stressors (Thornton et al. 2025).

In this work, we examine the mechanisms that contribute to rDNA homeostasis in fission yeast. We perform a comprehensive candidate approach in fission yeast to evaluate how different mutants associated with genome maintenance and heterochromatin affect homeostasis of the rDNA, identifying similarities as well as differences from those observed in other species. Additionally, we determine the consequences of having an engineered minimal rDNA array. We show that cells with a contracted rDNA are more sensitive to genotoxic drugs, and that many pathways that are involved in fork licensing, fork protection, DNA damage signaling, chromatin modifiers, and DNA repair mechanisms are essential to maintaining proper stability and homeostasis in the rDNA repeats. We find that constitutive DNA alkylation damage results in fork slowing, and the TLS and BER pathways contribute over time to a smaller rDNA array. This identifies the rDNA as a key genomic fragile site under conditions of replication fork stress.

## Materials and methods

### Cell growth and physiology

Fission yeast cell growth and physiology were matched to the previous lab protocol described in Forsburg and Rhind (2006) and Sabatinos and Forsburg (2010). Cell growth rate was calculated using OD 595 nm over a 13 h period from hours 12–24. Cell length was calculated using an observer drawn map of cell lengths in ImageJ-FIJI (Schindelin et al. 2012). Long-term growth in MMS was performed by serial dilution once each day for the number of days tested. The DNA extraction and rDNA quantification protocol is described below.

### Artificially contracted rDNA array

A strain with a TET inducible I-PpoI endonuclease originally developed by Sunder et al. (2012) was grown on 3 μM anhydrotetracycline-rich YES media plates for 5 d. Colonies of various sizes were taken and grown overnight in rich liquid YES media, and DNA was extracted via phenol-chloroform extraction. rDNA quantification was calculated as follows.

### Cell length

Cells were prepared as in Green et al. (2015). Medium for all live cell imaging was PMG-HULALA (PMG + Histidine, Uracil, Leucine, Adenine, Lysine, Arginine) (225 mg/L each) (Sabatinos and Forsburg, 2010). Cells were concentrated by a brief microfuge spin and applied to 2% agarose pads made from PMG + HULA and prepared on glass slides sealed with VaLaP (1/1/1 w/w/w vaseline/lanolin/paraffin). Images were taken at OD.44 for each strain. Static images were collected at room temperature, 22 °C. Images were acquired with a DeltaVision Core (Applied Precision, Issaquah, WA, USA) microscope using a 60× N.A. 1.4 PlanApo objective lens and a 12-bit Photometrics CoolSnap HQII CCD. The system x-y pixel size is 0.109 μm. The softWoRx v4.1 (Applied Precision, Issaquah, WA, USA) software was used for acquisition. Single brightfield images were acquired. Cell length was calculated using the built-in measuring feature in ImageJ-Fiji (Schindelin et al. 2012).

### Pulse field gel electrophoresis

Pulse field gels were run on a Bio-Rad CHEF-DR III with the following parameters: angle 106°, 1200–1800 se switching time, total time 72 h, temperature 14 °C, 2 V/cm^2^. The gel was .7% Mb agarose using 1x TAE buffer.

### rDNA mutant screen

Biological duplicate or more mutant strains were either grown at 32 °C for 6 d or 25 °C for 8 d (for temperature-sensitive mutants) in 5 mL of liquid-rich YES media. Strains were serially diluted once each day to maintain growth in log phase as much as possible. On the last day of growth, the 5 mL culture was spun down, and DNA was extracted via phenol-chloroform extraction as in Forsburg and Rhind (2006). DNA concentration was calculated via a Nanodrop 1000 spectrophotometer (Thermo Scientific). Aliquots of 20 ng/μL were made, and both samples were stored at −20 °C until used. Technical duplicates of each biological triplicate qPCR were done using iTaq universal SYBR green supermix (Biorad) and a CFX96 connect real-time PCR system (Biorad). Twenty microliter samples were run with a final concentration of 1 ng/μL. Standard curves with an *R*^2^ > 0.98 were used for relative quantification. Final values were calculated as 18 s/*act1* gene ratios. Primer sequences were developed using Primer-BLAST (National Institute for Biotechnology Information). Primers sequences used were 18sFWD 5′-ATT GGA GGG CAA GTC TGG TG-3′, 18sREV 5′-CAG TCG ACC AGG CTC AAA-3′, act1FWD 5′-TGC TAC GTC GCT TTG GAC TT-3′, act1REV 5′-GGA AAA GAG CTT CAG GGG CA-3′.

#### rRNA expression

Biological triplicate samples of 20%, 40%, and WT strains were grown at 32 °C overnight in 5 mL of rich YES media. RNA extraction was performed using the Qiagen RNeasy mini kit. cDNA was made using Bio-Rad iScript cDNA synthesis kit and primer-specific reverse transcription (18 s vs the housekeeping genes *act1* or *cdc2*, primer sequences shown below). DNA concentration was calculated via a Nanodrop 1000 spectrophotometer (Thermo Scientific). Aliquots of 20 ng/μL were made cDNA was stored at −20 °C until used. Technical duplicates of each biological triplicate qPCR were done using iTaq universal SYBR green supermix (Biorad) and a CFX96 connect real-time PCR system (Biorad). Twenty microliter samples were run with a final concentration of 1 ng/μL. Standard curves with an *R*^2^ > 0.98 and a PCR efficiency >90% were used for relative quantification. Final values were calculated as 18 s/*act1* and 18 s/*cdc2* gene expression ratios. Primers sequences used were 18sFWD 5′-ATT GGA GGG CAA GTC TGG TG-3′, 18sREV 5′-CAG TCG ACC AGG CTC AAA-3′, act1FWD 5′-TGC TAC GTC GCT TTG GAC TT-3′, act1REV 5′-GGA AAA GAG CTT CAG GGG CA-3′, cdc2FWD 5′- GGT GTC CCT TTG CGG AAC TA-3′, cdc2REV 5′- ACG CTC CAA ATA TCA ACC CCA-3′.

## Results

### Reduced copy number of rDNA leads to impaired cell growth and sensitivity to genotoxins.

**Fig. 1.**
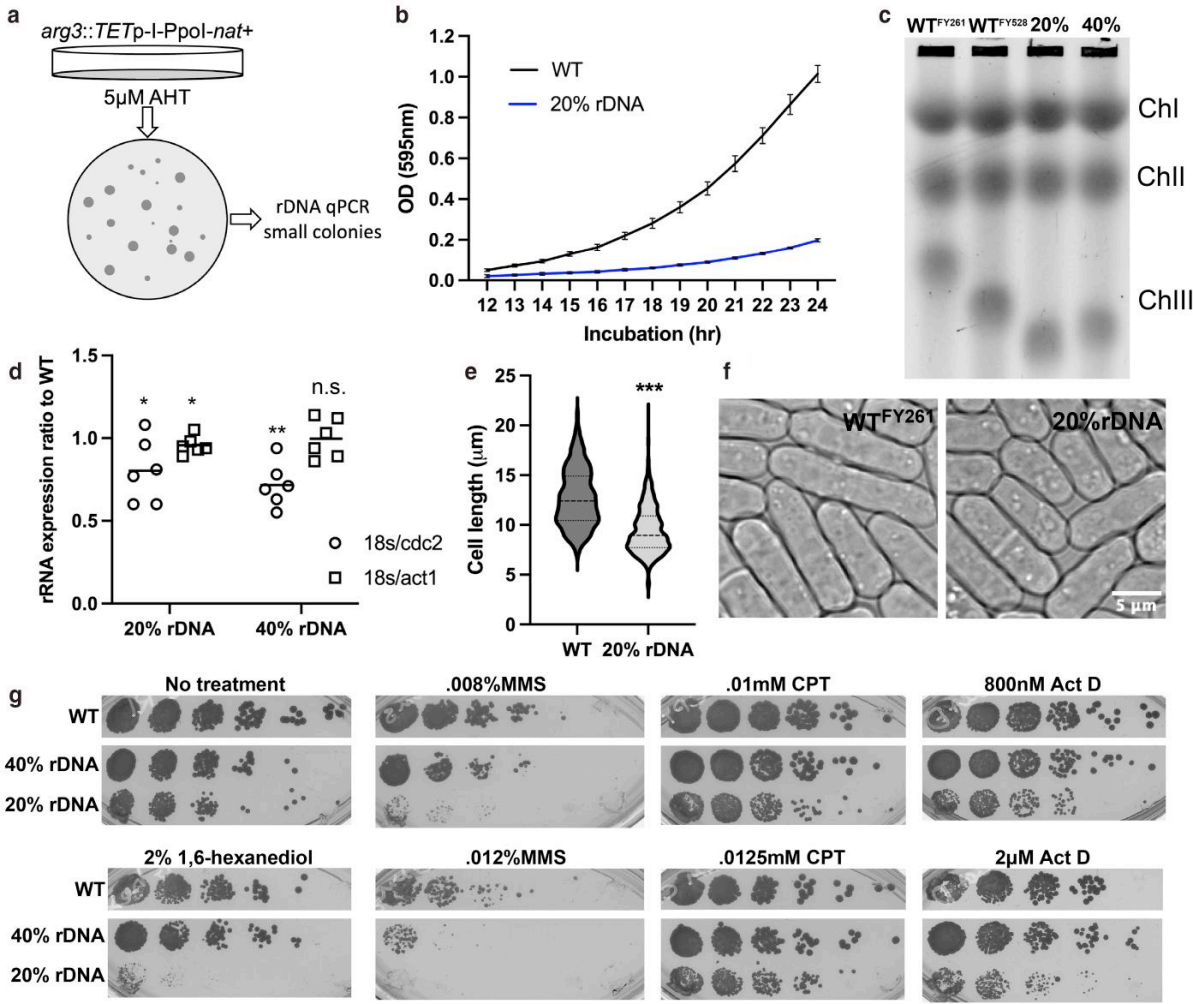
Low-copy number of rDNA leads to increased genome instability. a) Schematic of method to produce low-copy rDNA strains. b) Growth rate of 20% rDNA strain compared to WT using OD 595 nm. *n* = 3 c) PFGE of 2 WT strains compared to the artificially induced 20 and 40% rDNA strains. d) rRNA expression ratio of the artificially contracted 20 and 40% rDNA strains compared to WT261 using 2 housekeeping genes, act1 and cdc2 (*P < 0.05, **P < 0.01, ***P < 0.001, Mann–Whitney *U* test). e) Violin plot of cell lengths (µm) of WT vs 20% rDNA strain in rich YES media. Both OD = 0.44, *n* = 3 (*P same as above, Mann–Whitney *U* test). f) DIC reference images of WT and 20% rDNA strains. g) 1/5 Serial dilution stamping of WT, 40%, and 20% rDNA strains onto various drugs. WT 40 and 20% were all done on the same plate, but other strains on the plate were excluded, and thus, the images were cropped.

In order to evaluate the cellular response to a contracted rDNA array, we generated a minimal rDNA. We used a Tet inducible I-PpoI endonuclease to reduce the rDNA copy number of otherwise wild-type *S. pombe*. All eukaryotes contain an I-PpoI endonuclease recognition site in each rDNA repeat (Ellison and Vogt 1993). The site in *S. pombe* is located within the 25 s rRNA sequence and upon induction of I-PpoI, surviving cells contain an inserted T residue. This T insert into the rDNA has been deemed harmless and results in only a small increase in generation time (Sunder et al. 2012). Upon ahTet induction, we noticed a dramatic variability in colony size of survivors (Fig. 1 and Supplementary Fig. 1). We hypothesized that, due to induced double-strand breaks, the surviving strains could have a contraction in their arrays, and this could be the cause of the variation in colony size. We used qPCR to measure rDNA copy number by determining the ratio of 18S to the euchromatin gene *act1^+^* (as described below). The smallest of the colonies contained approximately 20% of the wild-type rDNA content.

We compared the rate of cell growth, overall cell length, and drug sensitivity of the minimal rDNA strain to the wild type. We observed a reduced growth rate of the 20% rDNA strain by comparing the OD_595_ over a 24 hr period (Fig. 1b). We also confirmed using pulsed field gel electrophoresis that the size of chromosome III (which contains the rDNA arrays) is proportional to the Q-PCR determined rDNA size in 20%, 40% and 2 different WT strains (Fig. 1c). To eliminate concerns that the decrease in growth rate were due to a decrease in rRNA transcription we also used qRT-PCR to quantify rRNA transcript levels compared to 2 housekeeping genes *act1* and *cdc2*. While levels are statistically significantly lower than WT rRNA, this effect is relatively modest (Fig. 1d). We observed that the 20% rDNA strain cells were smaller in size compared to wild type (Fig. 1e and f). When serially diluted onto plates containing the genotoxic drugs methyl methanesulfonate (MMS) or camptothecin (CPT), the transcription inhibitor actinomycin D (ActD), or the phase separation inhibitor 1,6-hexanediol, the 20% strain was more sensitive to all these drugs, with particular sensitivity to MMS and 1,6-hexanediol. In contrast, a strain with an intermediate 40% rDNA copy number ratio only shows increased sensitivity to higher doses of MMS (Fig. 1g).

### Candidate screen reveals pathways of rDNA copy number homeostasis.

**Fig. 2.**
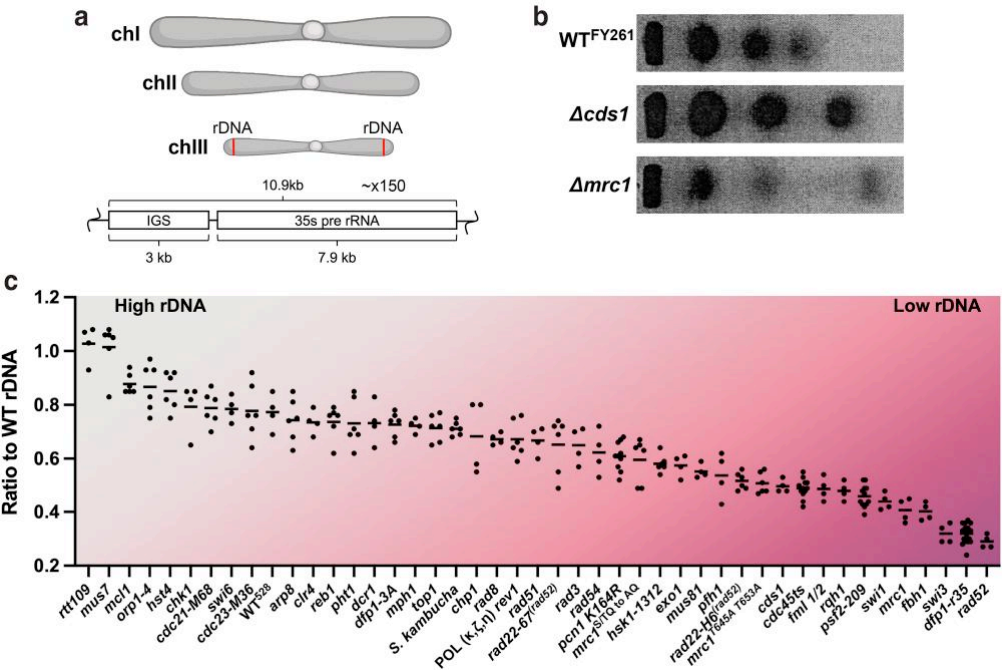
Targeted mutant screen reveals pathways of rDNA copy number homeostasis. a) Representation of the S. pombe chromsomes and structure of the ∼150, 10.9 kb rDNA repeats on chIII. b) PFGE of WT, and 2 example mutants to show correlation of chIII with rDNA size via qPCR. c) Selected examples from the full rDNA copy number screen that show the highest and lowest rDNA ratio to WTFY261. Dots represent all biological and technical replicates. Each biological replicate was done in technical duplicates.

To evaluate mechanisms that maintain rDNA homeostasis in *S. pomb*e, we developed a qPCR assay to compare rDNA between strains. For each strain, we determined the ratio of 18 s to *act1^+^* copy number. Since this analysis compares relative and not absolute copy number, we then divided the number compared to a wild-type *S. pombe* strain FY261, which has the same rDNA content as the original isolate wild-type strain 972 *h^−^* (data not shown). To confirm our results, we compared these ratio values to a previous PFGE of 3 representative strains, *Δcds1*, *Δmrc1,* and wild-type FY261 (Fig. 2a and b). We used a candidate screen to compare rDNA copy number using the 18 s/*act1* gene ratio compared to WT^FY261^ rDNA on a larger scale. The entire list of tested mutants is available in Supplementary Table 1. Selected mutant strains are shown in Fig. 2c. All mutants were tested with at least biological and technical duplicates due to technical variability in SYBRgreen-based qPCR and biological variability between clones. We observed a broad mid-range of copy number even in other wild-type strains compared to our arbitrary wild-type FY261 strain. Therefore, we focused our attention on strains at either end of the distribution with substantially increased or decreased copy number.

A mutation in the recombination mediator Δ*rad52* had the lowest rDNA content of any strain we examined. Surprisingly, other HR-associated mutants, such as *Δrad54, Δrad55, Δrad57,* and *Δrad51,* maintained an array size closer to wild type. We observed that a mutation in the helicase *Δfbh1* also contained very low rDNA. Since *Δfbh1* is a common spontaneous suppressor found in many *Δrad52* lab strains (Osman et al. 2005), it is possible that mutation of *Δfbh1* is responsible for the reduced rDNA levels in our *Δrad52* strain.

As observed previously, other mutants with very low rDNA content are those of the replication fork protection complex (FPC) *Δswi1, Δswi3,* and *Δmrc1* (Noguchi et al. 2004; Zech et al. 2015). The Swi1 and Swi3 proteins have been previously implicated in replication fork termination in the rDNA (Krings and Bastia 2004). In contrast, loss of the FPC-associated *mrc1* causes only a moderate reduction (26%) in RFB stalling by comparison (Zech et al. 2015). Mrc1 also plays a role in the activation of the DNA replication checkpoint kinase Cds1 (Tanaka and Russell 2004). We used 2 separations of function mutants Mrc1^T645A,T653A^ and Mrc1^S/TQ to AQ^ (Fig. 2c). Both of these alleles have mutations in the sites necessary for proper S phase damage signaling transduction while leaving the rest of the Mrc1 FPC functions as normal (Xu et al. 2006). These mutant alleles show a slightly larger rDNA array compared to full deletion *Δmrc1.* Deletion of the S phase checkpoint kinase Cds1 also showed a similar rDNA ratio to the Mrc1^T645A,T653A^ allele, indicating that loss of the replication checkpoint is not a contributor to rDNA instability. We also examined the *reb1Δ* mutant which disrupts fork termination in the rDNA (Sánchez-Gorostiaga et al. 2004), and it did not have an effect on rDNA copy number, suggesting it is not loss polar termination in an otherwise WT cell *per se* that is responsible for the reduced rDNA size.

**Fig. 3.**
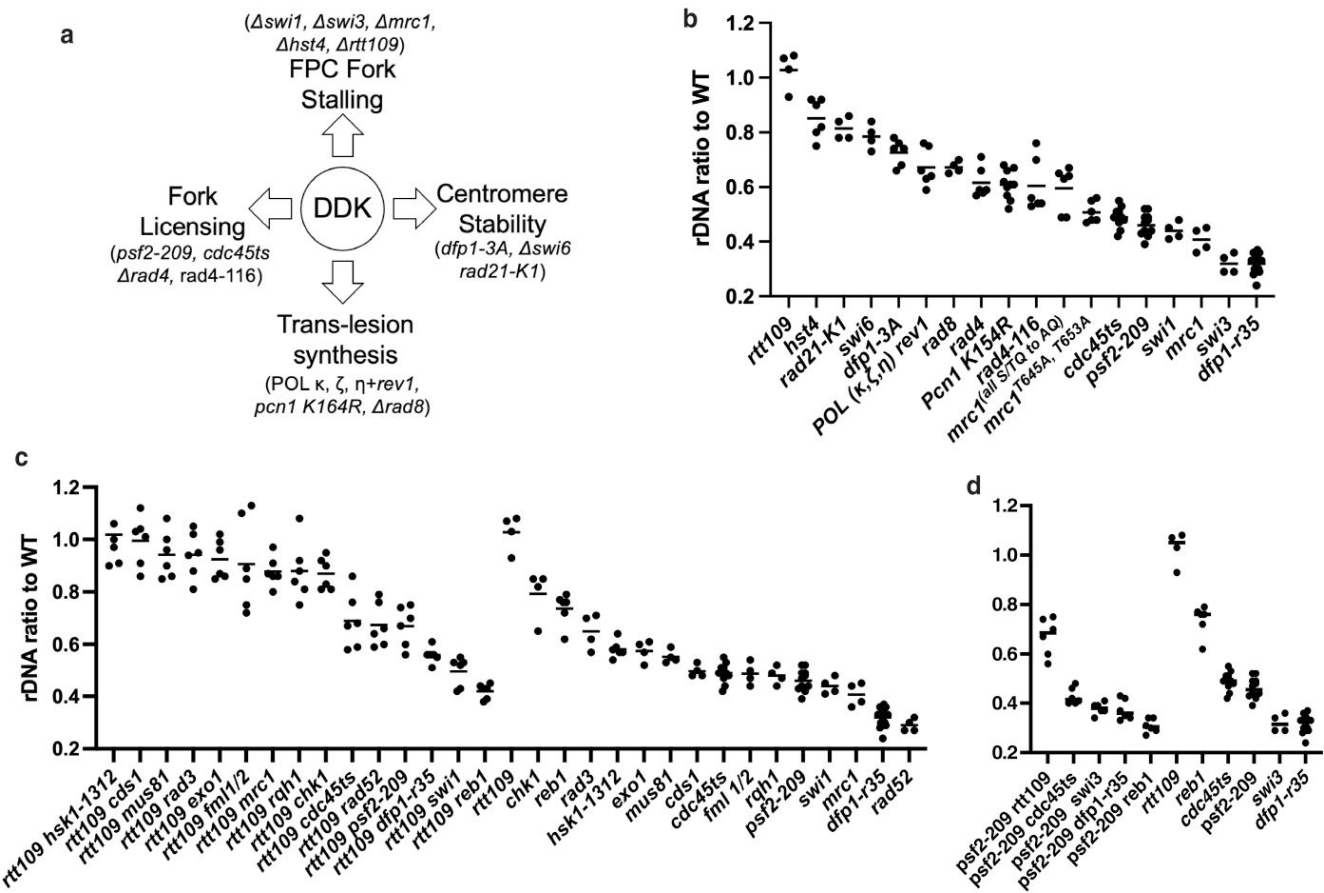
Pathways of rDNA homeostasis. a) rDNA contraction assay. Δswi1 and Δmrc1 were crossed with WT. Tetrads were dissected, and the spore with an expanded rDNA array and the deletion mutant were selected and grown for 8–9 d. b) DDK interacting pathways. c) Rtt109 interacting pathways and the mutants screened. d) Psf2 interacting pathways.

The FPC acts with the DNA replication kinase DDK in response to replication stress (Matsumoto et al. 2005; Sommariva et al. 2005; Shimmoto et al. 2009). DDK comprises a kinase Hsk1^Cdc7^ and a regulatory subunit Dfp1^Dbf4^ (Masai et al. 1995; Brown and Kelly 1999; Pasero et al. 1999; Snaith et al. 2000). We observed reduced rDNA array in C-terminal truncation mutant *dfp1-r35*, missing the last 25 amino acids of the Dfp1 protein (Dolan et al. 2010) (Figs. 2c and 3b). Similar results were observed in 2 other C-terminal truncation mutants, *dfp1(1-376)* and *dfp1(1-459*) (Fung et al. 2002) (Supplementary Table 2). The C-terminus of Dfp1 is required for full activation of Hsk1, proper MMS response, and is essential for proper meiosis (Fung et al. 2002; Dolan et al. 2010; Le et al. 2013). DDK plays many important roles in eukaryotic DNA replication and genome stability (Fig. 3a). We evaluated rDNA levels in multiple mutants in the pathways affected by DDK including centromere stability (*dfp1-3A*, Δ*swi6,rad21-K1*), fork licensing (*psf2-209, rad4, rad4-116, cdc45ts* (*sna41^goa1^*)), translesion synthesis (*pol* κ, ζ, η+*rev1, pcn1 K164R, rad8*), and replication fork stalling via the FPC (*Δswi1, Δswi3, Δmrc1, Δhst4, Δrtt109*) (Figs. 2c, and 3a, b). Only the FPC and fork licensing pathways shared a similarly low level of rDNA.

Fission yeast rDNA is partially heterochromatinized via H3K9 trimethylation and binding of HP1 orthologue Swi6 (Allshire et al. 1995; Cam et al. 2005). This mechanism is absent in *S. cerevisiae* (Rusche et al. 2003). We tested mutants with disruptions in essential heterochromatin proteins. Chp1 and Dcr1 are parts of the RITS complex associated with reestablishment of heterochromatin after replication by targeting the Clr4 histone methyltransferase (Petrie et al. 2005; Schalch et al. 2009). Swi6 is the HP1 orthologue protein that is responsible for H3K9me binding and condensation of heterochromatin domains (Allshire et al. 1995; Cam et al. 2005). Unexpectedly (and in contrast to *Drosophila;* Lawrence et al. 2004; Pontvianne et al. 2012), even without the essential functions of heterochromatin formation, we found that *Δswi6, Δdcr1, Δclr4,* and *Δchp1* all have a normal rDNA copy number.

Unlike H3K9me, another histone modification, H3K56ac is conserved between *S. pombe* and *S. cerevisiae* and is associated with DNA replication and DNA damage response (Hardy et al. 2019). The H3K56 histone acetyltransferase Rtt109 has been implicated in budding yeast rDNA homeostasis (Ide et al. 2013). Deletion *of S. cerevisiae rtt109* causes striking expansion of rDNA to 3 times that of the wild type (Ide et al. 2013). A similar expansion is observed in *S. cerevisiae Δmms22.* (Ide et al. 2013). In contrast to the magnitude of the effect in budding yeast, we observed only a modest increase in size of the rDNA arrays in *Δrtt109* or *Δmus7^mms22^* strains (Fig. 2c). Deletion of the histone deacetylase *Δhst4,* which antagonizes Rtt109, had no effect (Han et al. 2007; Yokoyama et al. 2007; Haldar and Kamakaka 2008).

We were curious how rapidly contraction occurs in a *swi1Δ* or *mrc1Δ* background. We crossed these strains with our designated WT strain and used tetrad dissection to identify clones in which a normal-sized rDNA was introduced into a *Δswi1* or *Δmrc1* background. Then, the strains in liquid media were sampled periodically for q-PCR. We found that over the space of approximately 60-70 generations, the rDNA contracted close to the size observed in our original mutant strain (Supplementary Fig. 2).

### Epistasis analysis of pathways of rDNA homeostasis

We investigated the phenotype of double mutants to assess the interaction between pathways that increase or decrease rDNA arrays. First, we examined double mutants with *Δrtt109* (Fig. 3b, c). Interestingly, the *Δrtt109 Δreb1* double mutant had a notably smaller rDNA array than either single mutant. This implies that Reb1-dependent termination at the RFB is required to stabilize the expanded array size seen with loss of H3K56ac. Loss of *Δrtt109* did not notably change the already shrunken array associated with *Δswi1,* but *Δrtt109 Δmrc1* had an increased array size. One explanation is that loss of FPC components Swi1/Swi3 leads to complete loss of fork stalling at rDNA RFB. However, loss of Mrc1 only leads to a 26% reduction in fork stalling at RFB (Zech et al. 2015). These results, along with the *Δrtt109 Δreb1,* further suggest that RFB termination is essential for the stability of the expanded rDNA array size seen in *Δrtt109*. Strikingly, a combination of *psf2-209 Δrtt109* or *cdc45ts Δrtt109* rescued the small array associated with *psf2-209 or cdc45ts* alone. This suggests that improper fork licensing leading to a contracted rDNA can be rescued by loss of H3K56ac.

Combination of *psf2-209* and *Δreb1* lead to pronounced contraction of the rDNA array size, also observed for *cdc45ts Δreb1* (Fig. 3d). The reduction goes from 46% in the *psf2-209* single mutant to 31% in the *psf2-209 Δreb1* and 49% in the *cdc45ts* single mutant to 38% in the *cdc45ts Δreb1* double mutant (Supplementary Fig. 3). This implies loss of RFB function and proper replication fork licensing may play separate role's in rDNA homeostasis and thus combination of loss of proper function leads to a combinatorial decrease in rDNA copy number.

### Alkylation damage causes a decrease in rDNA repeats.

**Fig. 4.**
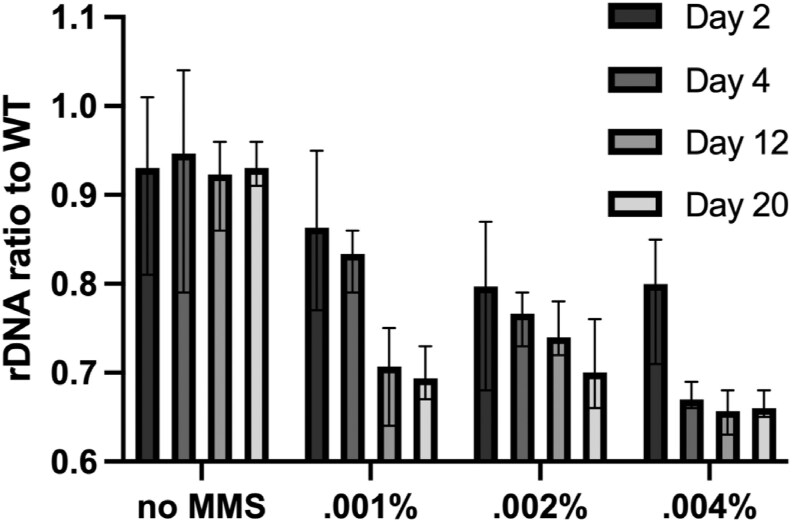
MMS stress causes rDNA array contraction. WT cells were grown in varying concentrations for a total of 20 d. Samples were taken at different intervals, and rDNA was quantified.

Methyl methanesulfonate (MMS) perturbs replication fork progression by causing the addition of a methyl group to mostly adenines and guanines (Beranek 1990; Lundin et al. 2005). Yeast cells deal with these adducts either via base excision repair pathways (BER) or via replication fork progression using error-prone translesion polymerases (Memisoglu and Samson 2000; Plosky et al. 2008). These processes interfere with transcription and replication machinery progression and must proceed despite the other competing processes. Since the rDNA completes replication late in S phase (Kim and Huberman 2001), further delay of the rDNA repeats due to ongoing repair may further sensitize larger arrays to greater instability. We hypothesized that long-term growth of cells in low concentrations of MMS would lead to a gradual contraction in rDNA copy number to cope with the added stress of completing rDNA replication in a timely manner. Our results show that even at.001% MMS over a 20-day period, there is a gradual decrease in rDNA copy number in WT cells (Fig. 4). The speed of rDNA copy number contraction was further exacerbated upon increasing the treatment to.004%. After 20 d even at the highest concentration, there was a stable reduction to about 65% of WT rDNA.

## Discussion

Repetitive DNA sequences contribute to chromosome fragile sites that are prone to DNA replication stress, DNA breakage, and genome rearrangements (Debatisse et al. 2012). The rDNA forms high-copy-number arrays potentially containing hundreds of repeats. The size of rDNA arrays in budding yeast has been linked to mechanisms maintaining genome stability (Ide et al. 2010; Kwan et al. 2016; Saka et al. 2016). Smaller array sizes are associated with increased instability.

Previously, we and others have shown that many *S. pombe* DNA replication mutants have an increased mobility of ChIII (Liang et al. 1999; Snaith et al. 2000; Liang and Forsburg 2001; Noguchi et al. 2012), which is consistent with a reduced rDNA array size and likely reflects increased double-strand breaks and recombination in these mutants. In the current study, we extended those observations to examine the stability of the rDNA repeats and mechanisms of rDNA homeostasis in fission yeast. Using a qPCR assay to determine repeat number, we showed that there is a modest variation in rDNA array size in different wild-type strains. Our control strain (FY261) falls on the upper end of this distribution, similar to the canonical wild-type 972*h*^−^. The results from the qPCR correlate with the size of chromosome III by PFGE. Although we did not independently assess the presence of extrachromosomal repeats (ERCs), these would be detected by the qPCR, allowing us to determine the total repeat copy number independent of chromosome size. We observed a modest variation in rDNA size in other wild-type strains, indicating some innate variation.

We examined the dynamics of rDNA repeats in wild-type cells. We generated a contracted rDNA array that is about 20% of wild-type levels. We find that this strain has a reduced growth rate, smaller ChIII, cell size, normal rRNA expression compared to WT, and increased sensitivity to several drugs. Compared to a strain with a 40% array size, we find that both of these are sensitive to the alkylating agent MMS. Work in budding yeast suggests that the size of rDNA arrays acts as a buffer for genome instability, and is also linked to replicative lifespan (Kobayashi 2011; Fine et al. 2019; Hotz et al. 2022). The sensitivity of our 20% strain is consistent with this model. We propose that the 20% strain defines the minimal functional rDNA size in unperturbed cells.

MMS creates DNA alkylation damage, which impairs replication fork progression, and is typically repaired by base excision repair (BER) or translesion synthesis pathways (Memisoglu and Samson 2000; Plosky et al. 2008). This damage can result in transcriptional and replication inhibition and thus must be repaired for proper cellular processes to proceed. In budding yeast cells with a contracted rDNA array, there is a much higher rDNA POLI occupancy on fewer rDNA repeats (Ide et al. 2010). Our data are consistent with this and suggest that competition between transcriptional machinery and repair machinery may sensitize the rDNA further in a strain with an already contracted rDNA array. We also observe that wild-type *S. pombe* contracts rDNA array size in response to constitutive low MMS-level treatment, though not as low as our minimal strain (Fig. 4). We suggest that this reflects errors in replication fork restart that lead to recombination events, with the normal array size providing a buffer against rearrangement.

It has been established that the nucleolus is phase separated (Weber and Brangwynne 2015; Feric et al. 2016), which can be disrupted by the aliphatic alcohol 1,6 hexanediol (Ribbeck and Görlich 2002; Kroschwald et al. 2015; Peskett et al. 2018; Ulianov et al. 2021). Previously, we showed that treatment with 1,6 hexanediol causes nucleolar fragmentation (Jones and Forsburg 2023). Here, we observed that the minimal rDNA array mutant is sensitive to low doses of the drug that wild-type cells can tolerate. This suggests that the minimal array mutant is exquisitely sensitive to any disruption of nucleolar architecture.

We performed a candidate screen of mutants implicated in genome stability to assess their effect on rDNA array size. We determined that MMS sensitivity correlates with reduced rDNA size, observing striking reductions in rDNA arrays in the MMS sensitive strains with disruptions in the fork protection complex (*Δswi1, Δswi3, Δmrc1),* DDK subunit *dfp1-r35,*genome stability proteins *Δfml1/2, Δrqh1,* and checkpoint kinase *Δcds1.* However, a number of mutants in our screen maintained a normal rDNA copy number despite MMS sensitivity, including checkpoint mutants *Δrad3* and *Δchk1,* recombination mutants *Δrad54* and *Δrad55,* and replisome mutant *mcm4-c106.* These results show that MMS sensitivity per se is not a determinant factor of rDNA array abnormality, but it is possible that there is a link between mutants that affect replication fork structure with reduced rDNA and MMS instability.

Instability of rDNA repeats has previously been for cells with mutations in the FPC proteins Swi1, Swi3, and Mrc1, which are implicated to various degrees in fork termination in the rDNA (Krings and Bastia 2004; Noguchi et al. 2004; Zech et al. 2015). However, the *reb1Δ* mutant which also disrupts fork termination in the rDNA (Sánchez-Gorostiaga et al. 2004) did not have an effect on rDNA copy number, suggesting it is not polar termination per se that is responsible for the reduced rDNA size. Interestingly, the effect of the FPC mutants is reasonably rapid, with a full size array contracting to the minimal size within about 60 generations.

The C-terminal truncation mutant *dfp1-r35* (Dolan et al. 2010) also reduces arrays size. DDK plays a distinct role in both fork licensing and FPC function. Dfp1 is responsible for targeting the Hsk1 kinase to phosphorylate MCM proteins during replication fork licensing. This allows for Cdc45 and GINS binding and thus facilitates fork licensing and replication firing (Dolan et al. 2004; Ilves et al. 2010; Aricthota and Haldar 2021). During fork progression, DDK also travels with the fork and is responsible for phosphorylating MCM proteins in response to genome damage, resulting in replication fork stalling via the FPC (Dolan et al. 2010). In budding yeast, association of FPC proteins Tof1–Csm3 (Swi1 and Swi3) with MCM proteins depends on DDK and thus abrogation of DDK leads to fork collapse instead of stabilized stalled forks (Matsumoto et al. 2005; Mohanty et al. 2006; Bastia et al. 2016). We propose that DDK plays a dual role in rDNA stability and rDNA copy number homeostasis through proper replication fork licensing as well as proper FPC function, and thus stabilizes stalled replication forks, which we suggest explains why the C-terminal *dfp1-r35* suffers a severe drop in rDNA copy number. We also observed reduced array size in replication fork licensing mutants such as *psf2-209* and *cdc45ts* (*sna41^goa1^*) (Dolan et al. 2004; Gómez et al. 2005), consistent with a model that impaired origin licensing and firing may additionally destabilize the rDNA.

While mutants with reduced rDNA arrays are MMS sensitive, we also observed MMS sensitivity in *Δrtt109* or *Δmus7,* which have a modestly expanded rDNA array. We speculate these are sensitive to MMS due to the inability to complete rDNA replication efficiently. Since replication of the rDNA repeats finishes late in S phase, completion of replication of a much larger array, along with replication delay due to repair functions, may further sensitize these strains (Kim and Huberman 2001). Notably, however, we observed that the increased array size in *Δrtt109* and *Δmus7* is much less dramatic in scale compared to that observed in the cognate mutants in *S. cerevisiae,* which generate ERCs (Ide et al. 2013). It is possible that ERC formation does not play the same role in *Δrtt109* in *S. pombe.* Since the rDNA arrays are at the chromosome termini in fission yeast, unequal sister exchange may be a more frequent mechanism, which would be consistent with the correlation between array number and chromosome III size. The expansion seen with *rtt109Δ* partially suppresses the full contraction observed with the FPC mutants. However, unexpectedly, the *rtt109Δ reb1Δ* double mutant is reduced considerably more than either single mutant, for reasons which are not clear.

Heterochromatin proteins regulate transcription in the rDNA (Thon and Verhein-Hansen 2000; Cam et al. 2005). We showed previously that the heterochromatic pericentromere repeats are prone to increased rearrangement in heterochromatin mutants that is enhanced by replication stress (Li et al. 2013). Previous studies have reported that proper heterochromatinization of silent rDNA repeats is essential for maintaining rDNA copy number homeostasis in plants (Lawrence et al. 2004; Pontvianne et al. 2012), and HP1 (Swi6) mutants in *Drosophila* have a successive contraction in their rDNA array over many generations (Aldrich and Maggert 2015). We were surprised that there was no change in rDNA copy number ratio in heterochromatin mutants *Δdcr1, Δclr4, Δchp1,* and Δ*swi6.* This may be because not all arrays are heterochromatinized (Cam et al. 2005). Also, there is an overlapping mechanism of heterochromatinization at telomeres in *S. pombe* that depends on H4 histone acetylation, regulated by Mst2 and Sir2. (Gómez et al. 2005). However, our analysis of *sir2Δ* showed a WT rDNA array. It remains possible that a similar overlapping system works in the telomere proximal rDNA, providing a redundancy in silencing and stability.

Taken together, this study highlights different mechanisms associated with maintaining rDNA homeostasis in fission yeast and identifies the minimal array size consistent with stability. There appears to be a key role associated with replisome activation and stability, with a central mediator being the DDK complex. Our data are consistent with the model that the rDNA array provides a buffer against genome instability through several overlapping pathways. However, these mechanisms can only withstand so much perturbation before contraction or expansion of the rDNA array results.

## Supplementary Material

jkag093_Supplementary_Data

## Data Availability

The authors affirm that all data necessary for confirming the conclusions of the article are present within the article, figures, and tables. All raw data is available upon request from the corresponding author. Supplemental material available at G3 online.

## References

[jkag093-B1] Akamatsu Y, Kobayashi T. 2015. The human RNA polymerase I transcription terminator complex acts as a replication fork barrier that coordinates the progress of replication with rRNA transcription activity. Mol Cell Biol. 35:1871–1881. 10.1128/MCB.01521-14. 25776556 PMC4405639

[jkag093-B2] Aldrich JC, Maggert KA. 2015. Transgenerational inheritance of diet-induced genome rearrangements in Drosophila. PLoS Genet. 11:e1005148. 10.1371/journal.pgen.1005148. 25885886 PMC4401788

[jkag093-B3] Allshire RC, Nimmo ER, Ekwall K, Javerzat JP, Cranston G. 1995. Mutations derepressing silent centromeric domains in fission yeast disrupt chromosome segregation. Genes Dev. 9:218–233. 10.1101/gad.9.2.218. 7851795

[jkag093-B4] Aricthota S, Haldar D. 2021. DDK/Hsk1 phosphorylates and targets fission yeast histone deacetylase Hst4 for degradation to stabilize stalled DNA replication forks. Elife. 10:e70787. 10.7554/eLife.70787. 34608864 PMC8565929

[jkag093-B5] Bastia D et al 2016. Phosphorylation of CMG helicase and Tof1 is required for programmed fork arrest. Proc Natl Acad Sci U S A. 113:E3639–E3648. 10.1073/pnas.1607552113. 27298353 PMC4932992

[jkag093-B6] Beranek DT . 1990. Distribution of methyl and ethyl adducts following alkylation with monofunctional alkylating agents. Mutat Res. 231:11–30. 10.1016/0027-5107(90)90173-2. 2195323

[jkag093-B7] Brewer BJ, Fangman WL. 1988. A replication fork barrier at the 30 end of yeast ribosomal RNA genes. Cell. 55:637–643. 10.1016/0092-8674(88)90222-X. 3052854

[jkag093-B8] Brown GW, Kelly TJ. 1999. Cell cycle regulation of Dfp1, an activator of the Hsk1 protein kinase. Proc Natl Acad Sci U S A. 96:8443–8448. 10.1073/pnas.96.15.8443. 10411894 PMC17535

[jkag093-B99] Carme P, Rutherford K, Bähler J, Mata J, Wood V. 2026. PomBase in 2026: expanding knowledge, modelling connections. Genetics. 232:iyag001. 10.1093/genetics/iyag001 41518600 PMC13050190

[jkag093-B9] Cam HP et al 2005. Comprehensive analysis of heterochromatin-and RNAi-mediated epigenetic control of the fission yeast genome. Nat Genet. 37:809–819. 10.1038/ng1602. 15976807

[jkag093-B10] Debatisse M, Le Tallec B, Letessier A, Dutrillaux B, Brison O. 2012. Common fragile sites: mechanisms of instability revisited. Trends Genet. 28:22–32. 10.1016/j.tig.2011.10.003. 22094264

[jkag093-B11] Dolan WP et al 2010. Fission yeast Hsk1 (Cdc7) kinase is required after replication initiation for induced mutagenesis and proper response to DNA alkylation damage. Genetics. 185:39–53. 10.1534/genetics.109.112284. 20176980 PMC2870973

[jkag093-B12] Dolan WP, Sherman DA, Forsburg SL. 2004. *Schizosaccharomyces pombe* replication protein Cdc45/Sna41 requires Hsk1/Cdc7 and Rad4/Cut5 for chromatin binding. Chromosoma. 113:145–156. 10.1007/s00412-004-0302-8. 15338237

[jkag093-B13] Ellison EL, Vogt VM. 1993. Interaction of the intron-encoded mobility endonuclease I-PpoI with its target site. Mol Cell Biol. 13:7531–7539. 10.1128/mcb.13.12.7531-7539.1993. 8246971 PMC364825

[jkag093-B14] Feric M, et al 2016. Coexisting liquid phases underlie nucleolar subcompartments. Cell. 165:1686–1697. 10.1016/j.cell.2016.04.047. 27212236 PMC5127388

[jkag093-B15] Fine RD, Maqani N, Li M, Franck E, Smith JS. 2019. Depletion of limiting rDNA structural complexes triggers chromosomal instability and replicative aging of *Saccharomyces cerevisiae*. Genetics. 212:75–91. 10.1534/genetics.119.302047. 30842210 PMC6499517

[jkag093-B16] Forsburg SL, Rhind N. 2006. Basic methods for fission yeast. Yeast. 23:173–183. https://10.1002/yea.1347. 16498704 10.1002/yea.1347 PMC5074380

[jkag093-B17] French SL, Osheim YN, Cioci F, Nomura M, Beyer AL. 2003. In exponentially growing *Saccharomyces cerevisiae* cells, rRNA synthesis is determined by the summed RNA polymerase I loading rate rather than by the number of active genes. Mol Cell Biol. 23:1558–1568. 10.1128/MCB.23.5.1558-1568.2003. 12588976 PMC151703

[jkag093-B18] Fung AD, Ou J, Bueler S, Brown GW. 2002. A conserved domain of *Schizosaccharomyces pombe* dfp1+ is uniquely required for chromosome stability following alkylation damage during S phase. Mol Cell Biol. 22:4477–4490. 10.1128/MCB.22.13.4477-4490.2002. 12052858 PMC133926

[jkag093-B19] Glover TW, Wilson TE, Arlt MF. 2017. Fragile sites in cancer: more than meets the eye. Nat Rev Cancer. 17:489–501. 10.1038/nrc.2017.52. 28740117 PMC5546318

[jkag093-B20] Gómez EB, Angeles VT, Forsburg SL. 2005. A screen for Schizosaccharomyces pombe mutants defective in rereplication identifies new alleles of rad4+, cut9+ and psf2 +. Genetics. 169:77–89. 10.1534/genetics.104.034231. 15466421 PMC1448876

[jkag093-B21] Good L, Intine RV, Nazar RN. 1997. Interdependence in the processing of ribosomal RNAs in Schizosaccharomyces pombe. J Mol Biol. 273:782–788. 10.1006/jmbi.1997.1351. 9367771

[jkag093-B22] Green MD, Sabatinos SA, Forsburg SL. 2015. Microscopy techniques to examine DNA replication in fission yeast. Methods Mol Biol. 1300:13–41. 25916703 10.1007/978-1-4939-2596-4_2

[jkag093-B23] Haldar D, Kamakaka RT. 2008. Schizosaccharomyces pombe Hst4 functions in DNA damage response by regulating histone H3 K56 acetylation. Eukaryot Cell. 7:800–813. 10.1128/EC.00379-07. 18344406 PMC2394969

[jkag093-B24] Han J et al 2007. Rtt109 acetylates histone H3 lysine 56 and functions in DNA replication. Science. 315:653–655. 10.1126/science.1133234. 17272723

[jkag093-B25] Hardy J et al 2019. Histone deposition promotes recombination-dependent replication at arrested forks. PLoS Genet. 15:e1008441. 10.1371/journal.pgen.1008441. 31584934 PMC6795475

[jkag093-B26] Hotz M et al 2022. rDNA array length is a major determinant of replicative lifespan in budding yeast. Proc Natl Acad Sci U S A. 119:e2119593119. 10.1073/pnas.2119593119. 35394872 PMC9169770

[jkag093-B27] Ide S, Miyazaki T, Maki H, Kobayashi T. 2010. Abundance of ribosomal RNA gene copies maintains genome integrity. Science. 327:693–696. 10.1126/science.1179044. 20133573

[jkag093-B28] Ide S, Saka K, Kobayashi T. 2013. Rtt109 prevents hyper-amplification of ribosomal RNA genes through histone modification in budding yeast. PLoS Genet. 9:e1003410. 10.1371/journal.pgen.1003410. 23593017 PMC3616922

[jkag093-B29] Ilves I, Petojevic T, Pesavento JJ, Botchan MR. 2010. Activation of the MCM2-7 helicase by association with Cdc45 and GINS proteins. Mol Cell. 37:247–258. 10.1016/j.molcel.2009.12.030. 20122406 PMC6396293

[jkag093-B30] Jaiswal R et al 2016. Functional architecture of the Reb1-Ter complex of *schizosaccharomyces pombe*. Proc Natl Acad Sci U S A. 113:E2267–E2276. 10.1073/pnas.1525465113. 27035982 PMC4843429

[jkag093-B31] Jones CE, Forsburg SL. 2023. Impact of 1, 6-hexanediol on Schizosaccharomyces pombe genome stability. G3 (Bethesda). 13:jkad123. 10.1093/g3journal/jkad123. 37284815 PMC10411564

[jkag093-B32] Kim SM, Huberman JA. 2001. Regulation of replication timing in fission yeast. EMBO J. 20:6115–6126. 10.1093/emboj/20.21.6115. 11689451 PMC125695

[jkag093-B33] Kobayashi T . 2003. The replication fork barrier site forms a unique structure with Fob1p and inhibits the replication fork. Mol Cell Biol. 23:9178–9188. 10.1128/MCB.23.24.9178-9188.2003. 14645529 PMC309713

[jkag093-B34] Kobayashi T . 2011. How does genome instability affect lifespan? Roles of rDNA and telomeres. Genes Cells. 16:617–624. 10.1111/j.1365-2443.2011.01519.x. 21605287 PMC3178783

[jkag093-B35] Kobayashi T, Ganley AR. 2005. Recombination regulation by transcription-induced cohesin dissociation in rDNA repeats. Science. 309:1581–1584. 10.1126/science.1116102. 16141077

[jkag093-B36] Krings G, Bastia D. 2004. swi1-and swi3-dependent and independent replication fork arrest at the ribosomal DNA of *Schizosaccharomyces pombe*. Proc Natl Acad Sci U S A. 101:14085–14090. 10.1073/pnas.0406037101. 15371597 PMC521093

[jkag093-B37] Krings G, Bastia D. 2005. Sap1p binds to Ter1 at the ribosomal DNA of *Schizosaccharomyces pombe* and causes polar replication fork arrest. J Biol Chem. 280:39135–39142. 10.1074/jbc.M508996200. 16195226

[jkag093-B38] Kroschwald S, et al 2015. Promiscuous interactions and protein disaggregases determine the material state of stress-inducible RNP granules. Elife. 4:e06807. 10.7554/eLife.06807. 26238190 PMC4522596

[jkag093-B39] Kwan EX et al 2023. Ribosomal DNA replication time coordinates completion of genome replication and anaphase in yeast. Cell Rep. 42:112161. 10.1016/j.celrep.2023.112161. 36842087 PMC10142053

[jkag093-B40] Kwan EX, Wang XS, Amemiya HM, Brewer BJ, Raghuraman MK. 2016. rDNA copy number variants are frequent passenger mutations in Saccharomyces cerevisiae deletion collections and de novo transformants. G3 (Bethesda). 6:2829–2838. 10.1534/g3.116.030296. 27449518 PMC5015940

[jkag093-B41] Lawrence RJ et al 2004. A concerted DNA methylation/histone methylation switch regulates rRNA gene dosage control and nucleolar dominance. Mol Cell. 13:599–609. 10.1016/S1097-2765(04)00064-4. 14992728

[jkag093-B42] Le AH, Mastro TL, Forsburg SL. 2013. The C-terminus of S. pombe DDK subunit Dfp1 is required for meiosis-specific transcription and cohesin cleavage. Biol Open. 2:728–738. 10.1242/bio.20135173. 23862021 PMC3711041

[jkag093-B43] Li PC et al 2013. Replication fork stability is essential for the maintenance of centromere integrity in the absence of heterochromatin. Cell Rep. 3:638–645. 10.1016/j.celrep.2013.02.007. 23478021 PMC3652564

[jkag093-B44] Liang DT, Forsburg SL. 2001. Characterization of *Schizosaccharomyces pombe* mcm7+ and cdc23+(MCM10) and interactions with replication checkpoints. Genetics. 159:471–486. 10.1093/genetics/159.2.471. 11606526 PMC1461838

[jkag093-B45] Liang DT, Hodson JA, Forsburg SL. 1999. Reduced dosage of a single fission yeast MCM protein causes genetic instability and S phase delay. J Cell Sci. 112:559–567. 10.1242/jcs.112.4.559. 9914167

[jkag093-B46] Lundin C et al 2005. Methyl methanesulfonate (MMS) produces heat-labile DNA damage but no detectable in vivo DNA double-strand breaks. Nucleic Acids Res. 33:3799–3811. 10.1093/nar/gki681. 16009812 PMC1174933

[jkag093-B47] Magdalou I, Lopez BS, Pasero P, Lambert SA. 2014. The causes of replication stress and their consequences on genome stability and cell fate. Semin Cell Dev Biol 30: 154–164. 24818779 10.1016/j.semcdb.2014.04.035

[jkag093-B48] Masai H, Miyake T, Arai KI. 1995. Hsk1+, a Schizosaccharomyces pombe gene related to Saccharomyces cerevisiae CDC7, is required for chromosomal replication. EMBO J. 14:3094–3104. 10.1002/j.1460-2075.1995.tb07312.x. 7621824 PMC394371

[jkag093-B49] Matsumoto S, Ogino K, Noguchi E, Russell P, Masai H. 2005. Hsk1-Dfp1/Him1, the Cdc7-Dbf4 kinase in *Schizosaccharomyces pombe*, associates with Swi1, a component of the replication fork protection complex. J Biol Chem. 280:42536–42542. 10.1074/jbc.M510575200. 16263721

[jkag093-B50] Memisoglu A, Samson L. 2000. Contribution of base excision repair, nucleotide excision repair, and DNA recombination to alkylation resistance of the fission yeast *Schizosaccharomyces pombe*. J Bacteriol. 182:2104–2112. 10.1128/JB.182.8.2104-2112.2000. 10735851 PMC111257

[jkag093-B51] Mohanty BK, Bairwa NK, Bastia D. 2006. The Tof1p–Csm3p protein complex counteracts the Rrm3p helicase to control replication termination of *Saccharomyces cerevisiae*. Proc Natl Acad Sci U S A. 103:897–902. 10.1073/pnas.0506540103. 16418273 PMC1347974

[jkag093-B52] Nelson JO, Watase GJ, Warsinger-Pepe N, Yamashita YM. 2019. Mechanisms of rDNA copy number maintenance. Trends Genet. 35:734–742. 10.1016/j.tig.2019.07.006. 31395390 PMC6744303

[jkag093-B53] Noguchi E, Noguchi C, McDonald WH, Yates JR III, Russell P. 2004. Swi1 and Swi3 are components of a replication fork protection complex in fission yeast. Mol Cell Biol. 24:8342–8355. 10.1128/MCB.24.19.8342-8355.2004. 15367656 PMC516732

[jkag093-B54] Noguchi C, Rapp JB, Skorobogatko YV, Bailey LD, Noguchi E. 2012. Swi1 associates with chromatin through the DDT domain and recruits Swi3 to preserve genomic integrity. PLoS One. 7:e43988. 10.1371/journal.pone.0043988. 22952839 PMC3431386

[jkag093-B55] Osman F, Dixon J, Barr AR, Whitby MC. 2005. The F-Box DNA helicase Fbh1 prevents Rhp51-dependent recombination without mediator proteins. Mol Cell Biol. 25:8084–8096. 10.1128/MCB.25.18.8084-8096.2005. 16135800 PMC1234329

[jkag093-B56] Pasero P, Duncker BP, Schwob E, Gasser SM. 1999. A role for the Cdc7 kinase regulatory subunit Dbf4p in the formation of initiation-competent origins of replication. Genes Dev. 13:2159–2176. 10.1101/gad.13.16.2159. 10465792 PMC316966

[jkag093-B57] Peskett TR, et al 2018. A liquid to solid phase transition underlying pathological huntingtin exon1 aggregation. Mol Cell. 70:588–601.e6. 10.1016/j.molcel.2018.04.007. 29754822 PMC5971205

[jkag093-B58] Petrie VJ, Wuitschick JD, Givens CD, Kosinski AM, Partridge JF. 2005. RNA interference (RNAi)-dependent and RNAi-independent association of the Chp1 chromodomain protein with distinct heterochromatic loci in fission yeast. Mol Cell Biol. 25:2331–2346. 10.1128/MCB.25.6.2331-2346.2005. 15743828 PMC1061622

[jkag093-B59] Plosky BS et al 2008. Eukaryotic Y-family polymerases bypass a 3-methyl-2′-deoxyadenosine analog in vitro and methyl methanesulfonate-induced DNA damage in vivo. Nucleic Acids Res. 36:2152–2162. 10.1093/nar/gkn058. 18281311 PMC2367705

[jkag093-B60] Pontvianne F et al 2012. Histone methyltransferases regulating rRNA gene dose and dosage control in Arabidopsis. Genes Dev. 26:945–957. 10.1101/gad.182865.111. 22549957 PMC3347792

[jkag093-B61] Ribbeck K, Görlich D. 2002. The permeability barrier of nuclear pore complexes appears to operate via hydrophobic exclusion. EMBO J. 21:2664–2671. 10.1093/emboj/21.11.2664. 12032079 PMC126029

[jkag093-B62] Rusche LN, Kirchmaier AL, Rine J. 2003. The establishment, inheritance, and function of silenced chromatin in Saccharomyces cerevisiae. Annu Rev Biochem. 72:481–516. 10.1146/annurev.biochem.72.121801.161547. 12676793

[jkag093-B63] Sabatinos SA, Forsburg SL. 2010. Molecular genetics of Schizosaccharomyces pombe. Meth Enzymol 470: 759–795. 10.1016/S0076-6879(10)70032-X 20946835

[jkag093-B64] Saka K, Takahashi A, Sasaki M, Kobayashi T. 2016. More than 10% of yeast genes are related to genome stability and influence cellular senescence via rDNA maintenance. Nucleic Acids Res. 44:4211–4221. 10.1093/nar/gkw110. 26912831 PMC4872092

[jkag093-B65] Sánchez-Gorostiaga A, López-Estrano C, Krimer DB, Schvartzman JB, Hernández P. 2004. Transcription termination factor reb1p causes two replication fork barriers at its cognate sites in fission yeast ribosomal DNA in vivo. Mol Cell Biol. 24:398–406. 10.1128/MCB.24.1.398-406.2004. 14673172 PMC303360

[jkag093-B66] Schalch T et al 2009. High-affinity binding of Chp1 chromodomain to K9 methylated histone H3 is required to establish centromeric heterochromatin. Mol Cell. 34:36–46. 10.1016/j.molcel.2009.02.024. 19362535 PMC2705653

[jkag093-B67] Schindelin J et al 2012. Fiji: an open-source platform for biological-image analysis. Nat Methods. 9:676–682. 10.1038/nmeth.2019. 22743772 PMC3855844

[jkag093-B68] Shimmoto M et al 2009. Interactions between Swi1-Swi3, Mrc1 and S phase kinase, Hsk1 may regulate cellular responses to stalled replication forks in fission yeast. Genes Cells. 14:669–682. 10.1111/j.1365-2443.2009.01300.x. 19422421 PMC2837079

[jkag093-B69] Shirahige K et al 1998. Regulation of DNA-replication origins during cell-cycle progression. Nature. 395:618–621. 10.1038/27007. 9783590

[jkag093-B70] Sinclair DA, Guarente L. 1997. Extrachromosomal rDNA circles—a cause of aging in yeast. Cell. 91:1033–1042. 10.1016/S0092-8674(00)80493-6. 9428525

[jkag093-B71] Singh SK, Sabatinos S, Forsburg S, Bastia D. 2010. Regulation of replication termination by Reb1 protein-mediated action at a distance. Cell. 142:868–878. 10.1016/j.cell.2010.08.013. 20850009 PMC2945231

[jkag093-B72] Snaith HA, Brown GW, Forsburg SL. 2000. Schizosaccharomyces pombe Hsk1p is a potential cds1p target required for genome integrity. Mol Cell Biol. 20:7922–7932 10.1128/.20.21.7922-7932.2000. 11027263 PMC86403

[jkag093-B73] Sommariva E et al 2005. Schizosaccharomyces pombe Swi1, Swi3, and Hsk1 are components of a novel S-phase response pathway to alkylation damage. Mol Cell Biol. 25:2770–2784. 10.1128/MCB.25.7.2770-2784.2005. 15767681 PMC1061638

[jkag093-B74] Srivastava R, Srivastava R, Ahn SH. 2016. The epigenetic pathways to ribosomal DNA silencing. Microbiol Mol Biol Rev. 80:545–563. 10.1128/MMBR.00005-16. 27250769 PMC4981667

[jkag093-B75] Sunder S, Greeson-Lott NT, Runge KW, Sanders SL. 2012. A new method to efficiently induce a site-specific double-strand break in the fission yeast Schizosaccharomyces pombe. Yeast. 29:275–291. 10.1002/yea.2908. 22674789 PMC3389596

[jkag093-B76] Tanaka K, Russell P. 2004. Cds1 phosphorylation by Rad3-Rad26 kinase is mediated by forkhead-associated domain interaction with Mrc1. J Biol Chem. 279:32079–32086. 10.1074/jbc.M404834200. 15173168

[jkag093-B77] Thon G, Verhein-Hansen J. 2000. Four chromo-domain proteins of Schizosaccharomyces pombe differentially repress transcription at various chromosomal locations. Genetics. 155:551–568. 10.1093/genetics/155.2.551. 10835380 PMC1461114

[jkag093-B78] Thornton K et al 2025. rDNA copy number variation affects yeast fitness in response to different environments. Genetics. 230:iyaf075. 10.1093/genetics/iyaf075. 40317179 PMC12239205

[jkag093-B79] Toda T, Nakaseko Y, Niwa O, Yanagida M. 1984. Mapping of rRNA genes by integration of hybrid plasmids in Schizosaccharomyces pombe. Curr Genet. 8:93–97. 10.1007/BF00420224. 24177582

[jkag093-B80] Tsang E, Carr AM. 2008. Replication fork arrest, recombination and the maintenance of ribosomal DNA stability. DNA Repair (Amst). 7:1613–1623. 10.1016/j.dnarep.2008.06.010. 18638573

[jkag093-B81] Ulianov SV, et al 2021. Suppression of liquid-liquid phase separation by 1,6-hexanediol partially compromises the 3D genome organization in living cells. Nucleic Acids Res. 49:10524–10541. 10.1093/nar/gkab249. 33836078 PMC8501969

[jkag093-B82] Weber SC, Brangwynne CP. 2015. Inverse size scaling of the nucleolus by a concentration-dependent phase transition. Curr Biol. 25:641–646. 10.1016/j.cub.2015.01.012. 25702583 PMC4348177

[jkag093-B83] Xu Y, Davenport M, Kelly TJ. 2006. Two-stage mechanism for activation of the DNA replication checkpoint kinase Cds1 in fission yeast. Genes Dev. 20:990–1003. 10.1101/gad.1406706. 16618806 PMC1472306

[jkag093-B84] Yokoyama M, Inoue H, Ishii C, Murakami Y. 2007. The novel gene mus7+ is involved in the repair of replication-associated DNA damage in fission yeast. DNA Repair (Amst). 6:770–780. 10.1016/j.dnarep.2007.01.005. 17307401

[jkag093-B85] Zech J, Godfrey EL, Masai H, Hartsuiker E, Dalgaard JZ. 2015. The DNA-binding domain of S. pombe Mrc1 (claspin) acts to enhance stalling at replication barriers. PLoS One. 10:e0132595. 10.1371/journal.pone.0132595. 26201080 PMC4511789

[jkag093-B86] Zeman MK, Cimprich KA. 2014. Causes and consequences of replication stress. Nat Cell Biol. 16:2–9. 10.1038/ncb2897. 24366029 PMC4354890

