# Supplementary material for: Pathways of rDNA copy number homeostasis in Schizosaccharomyces pombe
Source: G3 (Bethesda). 2026 Apr 28;16(6):jkag093. doi: 10.1093/g3journal/jkag093 (PMC13232510; doi:10.1093/g3journal/jkag093)
Supplement: jkag093_Supplementary_Data [file jkag093_supplementary_data.zip › Supplemental_Figure_1_G3-2026-406616.pdf]

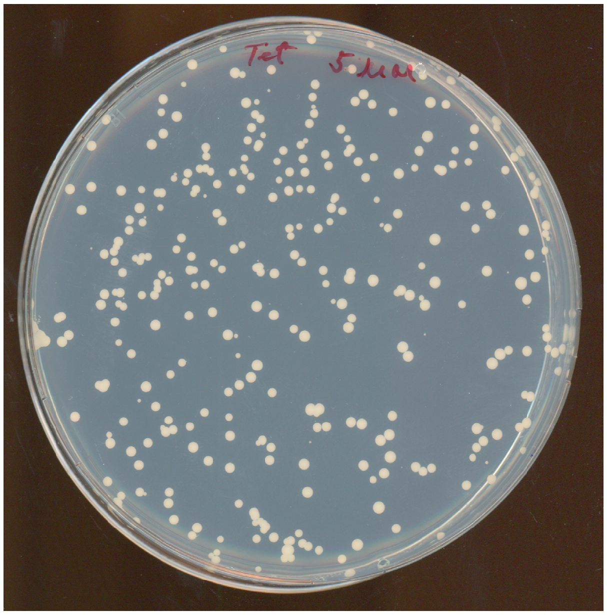

Supplemental Figure 1. Colony size difference
Colony size variability after iPpoI induction. Small colonies and large colonies were chosen and 18s/actin qPCR was performed showing that the smaller colonies contained a smaller rDNA array.
