# Supplementary material for: Pathways of rDNA copy number homeostasis in Schizosaccharomyces pombe
Source: G3 (Bethesda). 2026 Apr 28;16(6):jkag093. doi: 10.1093/g3journal/jkag093 (PMC13232510; doi:10.1093/g3journal/jkag093)
Supplement: jkag093_Supplementary_Data [file jkag093_supplementary_data.zip › Supplemental_Figure_2_G3-2026-406616.pdf]

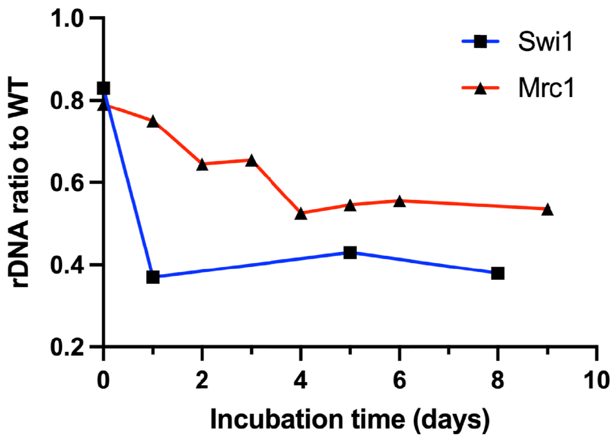

Supplemental Figure 2. rDNA contraction in FPC mutants

FPC mutants were crossed with a WT rDNA strain. Mutant tetrads with a WT rDNA were selected and tracked over time.
