# Supplementary material for: Pathways of rDNA copy number homeostasis in Schizosaccharomyces pombe
Source: G3 (Bethesda). 2026 Apr 28;16(6):jkag093. doi: 10.1093/g3journal/jkag093 (PMC13232510; doi:10.1093/g3journal/jkag093)
Supplement: jkag093_Supplementary_Data [file jkag093_supplementary_data.zip › Supplemental_Figure_3_G3-2026-406616.pdf]

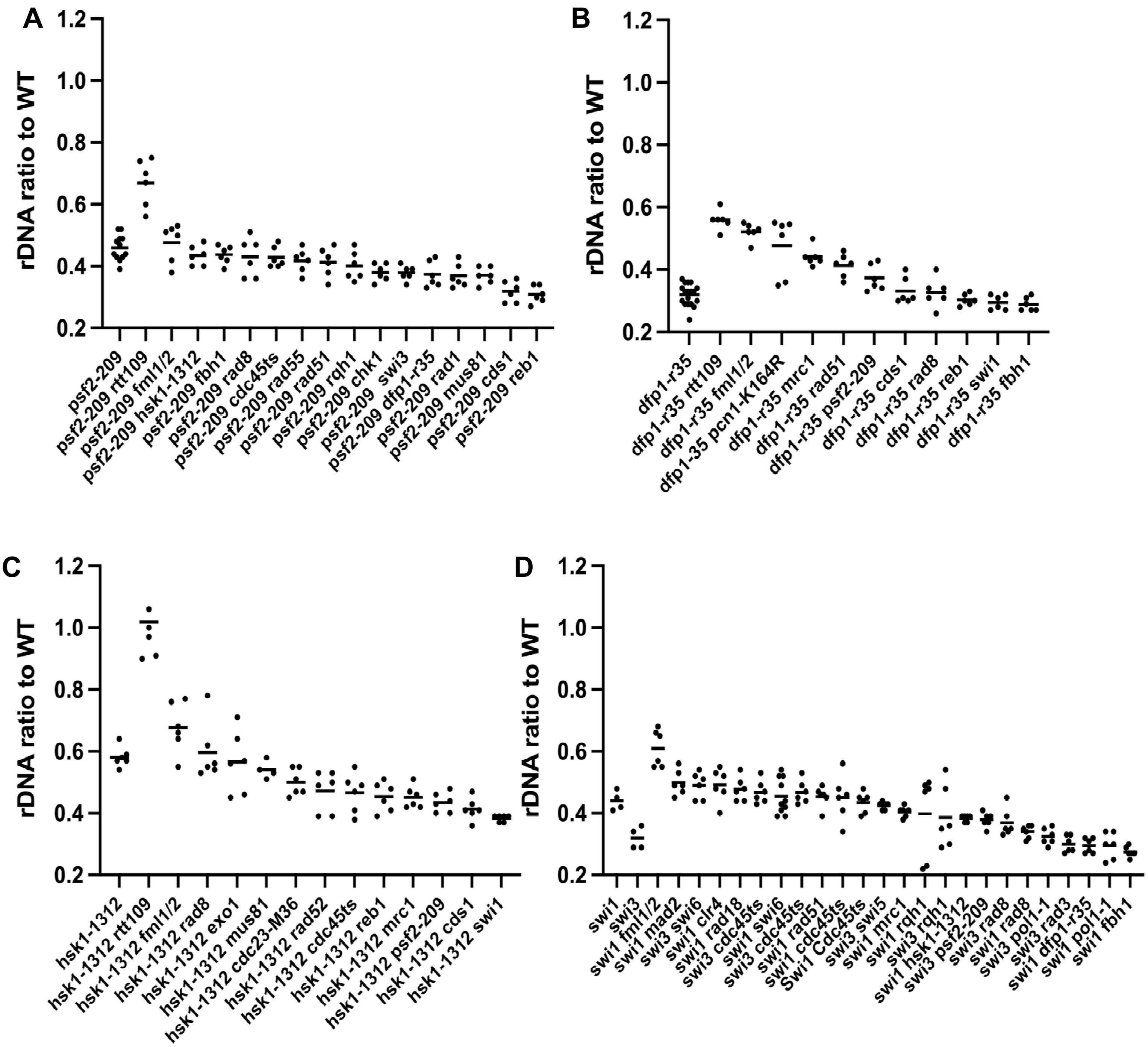

Supplemental Figure 3. Various selected double mutants

A-D Selected double mutant combinations of *psf2-209*, *dfp2-r35*, *hsk1-1312*, and FPC mutants
