# Supplementary material for: Pathways of rDNA copy number homeostasis in Schizosaccharomyces pombe
Source: G3 (Bethesda). 2026 Apr 28;16(6):jkag093. doi: 10.1093/g3journal/jkag093 (PMC13232510; doi:10.1093/g3journal/jkag093)
Supplement: jkag093_Supplementary_Data [file jkag093_supplementary_data.zip › Supplemental_Material_Legends_G3-2026-406616.docx]

**Supplemental Figure 1. Colony size difference**

Colony size variability after iPpoI induction. Small colonies and large colonies were chosen and 18s/actin qPCR was performed showing that the smaller colonies contained a smaller rDNA array.

**Supplemental Figure 2. rDNA contraction in FPC mutants**

FPC mutants were crossed with a WT rDNA strain. Mutant tetrads with a WT rDNA were selected and tracked over time.

**Supplemental Figure 3. Various selected double mutants**

A-D Selected double mutant combinations of psf2-209, dfp2-r35, hsk1-1312, and FPC mutants.

**Supplemental Table 1. Strain list**

This strain list shows all strains used in experiments. Strain numbers are lab associated strain numbers for record keeping.

**Supplemental Table 2. Raw relative qPCR values for Single and Double Mutants**

qPCR values calculated as 18s/act1 relative to WT 18s/act1
